# Psychological richness can enhance innovative behavior of Chinese college students: the chain mediating role of cognitive flexibility and creative self-efficacy

**DOI:** 10.3389/fpsyg.2025.1630873

**Published:** 2025-08-07

**Authors:** Peilin Yu, Aiyao He, Qinyan Wang, Yutang Li, Si Hu, Yuwei Gao

**Affiliations:** ^1^School of Education, Hunan University of Science and Technology, Xiangtan, China; ^2^Faculty of Psychology, Southwest University, Chongqing, China; ^3^College of Continuing Education, Hunan Applied Technology University, Changde, China; ^4^Student Affairs Department of the Party Committee, Yan'an University, Yan'an, China

**Keywords:** psychological richness, innovative behavior, cognitive flexibility, creative self-efficacy, college student

## Abstract

To investigate the relationship between psychological richness and innovative behavior among Chinese college students, as well as the roles of cognitive flexibility and creative self-efficacy in this relationship, this study employed the Psychologically Rich Life Questionnaire, Cognitive Flexibility Scale, Creative Self-Efficacy Scale, and Innovative Behavior Scale to survey 669 Chinese college students. The results show that: (1) Psychological richness positively predicts innovative behavior; (2) Cognitive flexibility and creative self-efficacy, respectively, play a partial mediating role between psychological richness and innovative behavior; (3) Cognitive flexibility and creative self-efficacy play a chain mediating role between psychological richness and innovative behavior. The findings enrich the research results in the field of innovative behavior among Chinese college students and provide enlightenment for the cultivation of innovative talents in Chinese higher education.

## 1 Introduction

Adolescence is a critical stage for the formation and development of innovative behavior, and the innovative behavior of college students even to some extent determines the country's economic development potential and international competitiveness (Li et al., 2019), therefore, in recent years, the cultivation of innovative talents has also received high attention from government departments (Central Committee of the Communist Party of China State and Council, 2019), and universities, as important bases for talent cultivation (Mei et al., 2015), are the main battlefield for cultivating innovative talents (Yu and Hou, 2004). Innovative behavior refers to all behaviors in the process from the generation of innovative ideas to the realization of ideas (Wang et al., 2022a; Scott and Bruce, 1994), which not only has a positive impact on students' mental health development (Yu and Zhang, 2019), but also an important driving force to promote the sustainable development of society (Aboobaker and Zakkariya, 2019). However, past studies have mainly focused on the innovative behavior of employees (Salam and Senin, 2022) and lacked the exploration of the factors influencing the innovative behavior of college students (Alt et al., 2023). Therefore, exploring the factors that influence college students' innovative behavior and their underlying mechanisms has certain theoretical value and practical significance.

Previous studies have explored the key role of individual factors in the development of college students' innovative behaviors (Zheng, 2023). Researchers generally believe that happiness is an important positive psychological factor promoting the development of college students' innovative behaviors (Agaoglu et al., 2025). Both a happy life (Diener et al., 2018) and a meaningful life (Ryff, 1989) have been proven to positively predict the innovative behavior of college students (Huang and Zhang, 2024; Ma et al., 2024). As positive psychology continues to evolve, (Oishi et al. 2019) have proposed an alternative form of happy living from a perspective that focuses on cognitive development: psychological richness. This new way of living a happy life complements the dualism of the traditional view of happiness and expands the research perspective of positive psychology. It is not a simple experience of diversity, but a psychological trait formed after people experience strong emotions and realize the transformation of cognitive perspective in a diversified life (Oishi and Westgate, 2022). Social Cognitive Theory states (Bandura, 1986) that psychological richness can be used as an individual factor that determines the development of their behavior to some extent. It has been argued that starting with psychological richness is a good start to promote innovative behavior in individuals (Oishi and Westgate, 2025).

However, previous studies have focused on the antecedent variables that cause psychological richness, and less on psychological richness as an antecedent variable to explore what results it may bring. Compared with the two traditional theoretical paradigms of hedonism and eudaimonic wellbeing, the psychologically rich life shows significant characteristics of long-term and easy availability (Liu et al., 2025). Individuals who obtain wellbeing with a traditional conception of happiness often have to pay more, which makes the effect of wellbeing on innovative behavior subject to different factors (Li et al., 2021; Oishi and Westgate, 2022). In contrast to traditional wellbeing, psychological richness enables individuals to have a favorable psychological experience and internal motivation in a more direct and long-lasting way. This may be more conducive to the positive development of an individual's innovative behavior (Liu et al., 2025).

For college students, cognitive flexibility and creative self-efficacy are important factors that contribute to the development of their innovative behavior (Ford, 1996; Zhou et al., 2021), while it has been confirmed that cognitive flexibility and creative self-efficacy may play a key role in the relationship between psychological factors and innovative behavior (Dreu et al., 2011; Pajares and Graham, 1999). On the one hand, according to the Constructionist Theory, college students actively construct new knowledge frameworks when interacting with new things (Raskin, 2011), which promotes the development of their cognitive abilities (Wei and Yu, 2024); On the other hand, self-efficacy theory (Bandura, 1977) suggests that creative self-efficacy is derived from innovation-related performance accomplishment, and that the more psychologically enriched individuals are, the more likely them have had experience with accomplishing an innovative task, and that such performance accomplishment can help to increase their corresponding self-efficacy. This shows that cognitive flexibility and creative self-efficacy may be the intermediary factors between psychological richness and innovative behavior.

Despite the critical importance of innovative behavior to the development of college students and the attention paid to psychological richness as an emerging dimension of wellbeing, however, few studies have simultaneously focused on the relationship between psychological richness, an emerging dimension of wellbeing, and college students' innovative behavior. Cognitive flexibility and creative self-efficacy have been identified as important sources of innovative behavior, but their relationship with psychological richness and how they work together with psychological richness to influence the process of innovative behavior has not been fully explored theoretically and empirically. Therefore, this study, based on the social cognitive theoretical framework and constructivist theory, intends to investigate the relationship between psychological richness and college students' innovative behavior and its internal mechanism in the context of Chinese culture, which not only helps to promote the application of social cognitive theory and constructivist theory in the field of positive psychology, but also provides new ideas and valuable scientific basis for the cultivation of Chinese college students' innovative behavior.

## 2 Literature review and hypothesis development

### 2.1 Psychological richness and innovative behavior of college students

Psychological richness refers to the psychological traits formed by individuals through diverse, novel, interesting, and complex life experiences, after experiencing strong emotional fluctuations and cognitive perspective changes. It is not only a temporary state of mind, but also an ongoing lifestyle choice. A life of many life experiences characterized by novelty, fun, etc., is a psychologically rich life (Besser and Oishi, 2020; Oishi and Westgate, 2022; Wei and Yu, 2024). From the perspective of social cognitive theory, when faced with complex, novel, and unknown scenarios, people think about solutions to problems, and during this process, cognitive perspectives change significantly, which can help people make multiple attributions about the problem (Oishi et al., 2024), and diverse and complex perspectives are conducive to innovation (Lin and Chang, 2024), as (Selznick et al. 2021) found that diverse and complex learning methods can help college students perform better in innovative behaviors. Therefore, according to social cognitive theory, psychological richness as an individual factor may have an impact on innovative behavior as a behavioral factor. Psychologically rich life is filled with the unknown, and this uncertainty stimulates an individual's curiosity, prompting them to actively explore uncharted territory and seek out novel experiences, which makes psychologically rich individuals tend to have greater cognitive needs (Besser and Oishi, 2020) and a desire to engage in novel, interesting, and challenging activities to enrich their lives (Oishi et al., 2019). For example, college students with higher levels of psychological richness are more likely to choose challenging courses (Oishi and Westgate, 2022), which can promote the development of their innovative abilities (Selznick et al., 2022) and lead to more innovative behaviors (Guo and Deng, 2020). Meanwhile, the relationship is also consistent with Self-Determination Theory, where the pursuit of a psychologically rich life may satisfy an individual's need for autonomy (Ryan and Deci, 2000), which in turn stimulates innovative behavior (Kwon and Kim, 2025). For example, (Oishi et al. 2019) found that there was a significant positive correlation between psychological richness and autonomy, and autonomy was conducive to the development of college students' innovative behavior (Al-Mamary and Alshallaqi, 2022). Thus, this study puts forward the hypothesis H1: Psychological richness can positively predict the innovative behavior of college students.

### 2.2 The mediating role of cognitive flexibility

Cognitive flexibility is the cognitive ability of an individual to flexibly adjust coping strategies in the face of changing circumstances (Dennis and Vander Wal, 2010; Martin and Rubin, 1995). Psychologically rich individuals like to pursue novel experiences and need to give up old mental representations to cope with new experiences, to achieve cognitive reconstruction (Oishi and Westgate, 2022). Therefore, psychological richness can promote cognitive development (Wei and Yu, 2024). For example, (Yu and Su 2015) showed that participating in novel and complex learning activities can help college students have a richer cognitive perspective and show stronger cognitive flexibility. This is consistent with the Constructionist Theory that psychologically rich college students are enthusiastic about participating in novel and complex activities. In the process of interacting with the surrounding environment, they will integrate new knowledge with existing cognitive frameworks, construct new cognitive structures, and promote the development of cognitive flexibility (Wang, 2005). At the same time, cognitive flexibility is an important factor in predicting the innovative behavior of college students. Related research has found that there is a positive correlation between cognitive flexibility and innovative behavior (Yang et al., 2021), and training college students' thinking flexibility can effectively cultivate their innovative ability (Ruscio and Amabile, 1999). Further research shows that college students' cognitive flexibility significantly positively predicts their innovative abilities (Zhou et al., 2021). A longitudinal follow-up study also shows that cultivating the cognitive flexibility of college students can significantly enhance their innovative behaviors (Büning et al., 2021). In addition, the process of psychological richness is often accompanied by intense emotional fluctuations (Oishi and Westgate, 2022), in the process of emotion affecting innovation behavior, cognitive flexibility plays an important role (Dreu et al., 2011), The dual pathway to creativity model proposed by (De Dreu et al. 2008) believes that the activation of emotional state affects college students' willingness to innovate through the mediating role of cognitive flexibility. This indicates that psychological richness may influence college students' willingness to innovate through the mediating role of cognitive flexibility. Thus, this study puts forward the hypothesis H2: Cognitive flexibility may play a mediating role between psychological richness and college students' innovative behavior.

### 2.3 The mediating role of creative self-efficacy

Self-efficacy refers to an individual's belief that they can complete a certain activity (Bandura, 1977), and creative self-efficacy is a special form of self-efficacy (Iqbal et al., 2022). It is defined as the belief that a person can produce creative results (Tierney and Farmer, 2002). Psychologically rich life is filled with a variety of novel, complex, and innovative challenges (Oishi and Westgate, 2022), and success in dealing with these challenges results in the accumulation of performance accomplishments, as indicated by self-efficacy theory that performance accomplishment is an important source of innovative self-efficacy, and therefore, psychological enrichment may have a significant impact on innovative self-efficacy. Some studies have found that there is a significant positive correlation between psychological richness and creative self-efficacy (Wang et al., 2022b). Psychological richness not only provides an experiential source of creative self-efficacy (Bandura, 1977) but also helps individuals to strengthen creative self-efficacy through the process of cognitive restructuring (Bandura, 1989). Further research has found that cultivating students' psychological richness can effectively enhance their creative self-efficacy (Keyser and Barling, 1981), as (Wang et al. 2024) found that teaching methods that facilitate cognitive reconstruction can significantly enhance college students' creative self-efficacy. Hence, psychological richness may contribute to enhancements that have a significant impact on college students' creative self-efficacy. Secondly, creative self-efficacy is an important predictor of innovative behavior (Iqbal et al., 2022), and the belief that they can innovate can promote the development of college students' innovative behavior (Ford, 1996). Relevant empirical studies have found that creative self-efficacy can positively predict innovative behavior (Tierney and Farmer, 2002), which is also supported by longitudinal tracking surveys (Afsar and Masood, 2018). Furthermore, according to self-efficacy theory, self-efficacy can mediate the effects of psychological factors on innovative behavior (Pajares and Graham, 1999). meaning that psychological changes will change the intensity of self-efficacy, which will lead to changes in innovative behavior. Thus, this study proposes hypothesis H3: Creative self-efficacy may mediate the relationship between psychological richness and college students' innovative behavior.

### 2.4 The chain mediating role of cognitive flexibility and creative self-efficacy

College students with high cognitive flexibility are more likely to achieve excellent academic performance (Gökçe and Güner, 2024), and academic performance can enhance self-efficacy (Kurtovic et al., 2019). This suggests that cognitive flexibility may contribute to the development of self-efficacy (Liu et al., 2024). Some studies have found that there is a certain predictive relationship between cognitive flexibility and creative self-efficacy (Yu et al., 2023); cognitive flexibility can significantly positively predict college students' creative self-efficacy (Mishra and Singh, 2024). Therefore, psychological richness may enhance creative self-efficacy by promoting the development of cognitive flexibility (Mortimer et al., 2012; Orakci and Khalili, 2025), thereby influencing innovation behavior. On the one hand, according to the Constructionist Theory, a psychologically rich life is conducive to the cognitive reconstruction of college students (Oishi and Westgate, 2022) and promotes the development of cognitive ability (Oishi et al., 2024). On the other hand, based on the theory of self-efficacy, creative self-efficacy has been proved to be an important mediator between cognitive flexibility and behavioral performance (Chin and Kameoka, 2002). The improvement of cognitive ability can enhance self-efficacy (Bandura, 1989) and then affect innovative behavior (Ford, 1996); that is, cognitive flexibility can affect innovative behavior through the role of creative self-efficacy (Yu et al., 2023). Thus, this study proposes Hypothesis H4: Cognitive flexibility and creative self-efficacy may play a chain mediating role between psychological richness and college students' innovative behavior.

**Figure 1:**
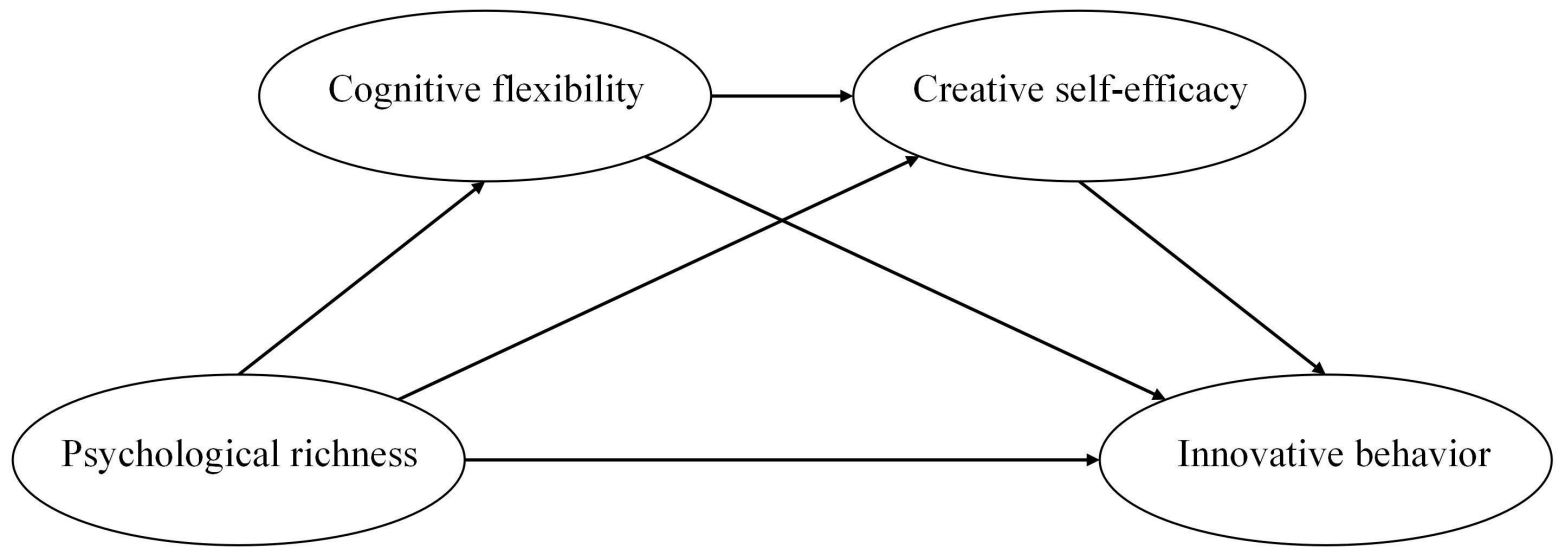
The hypothesized model.

In summary, this study intends to construct a chain mediation model (as shown in Figure 1) to explore the impact of psychological richness on Chinese college students' innovative behavior, and to examine the independent and chain mediation effects of cognitive flexibility and creative self-efficacy. Social cognitive theory emphasizes that individual factors are subjective, and within this theoretical framework, psychological richness, cognitive flexibility, and innovation self-efficacy are all individual factors, suggesting that their measurement needs to be achieved through subjective self-reports. Meanwhile, innovative behaviors contain multiple stages that require participants to look back into the past, which also needs to be measured through the self-report method. Therefore, the self-report method was used in this study to measure the research variables.

## 3 Methods

### 3.1 Procedures and participants

The survey is consistent with the principles of the Declaration of Helsinki and approved by the Ethics Committee of Hunan University of Science and Technology. In this study, 752 college students from three universities in Hunan and Shaanxi were selected as the participants by convenient sampling. The class teacher distributed electronic questionnaires through Wenjuanxing's online survey platform in the classroom for testing. According to the answers to the quality test questions, invalid samples were eliminated. The two quality test questions required the subjects to choose “6” and “2”. If participants did not answer as required, the data would be eliminated. Finally, 669 valid samples were obtained, with an effective rate of 88.96 %. The age of the subjects ranged from 17 to 26, including 146 boys (21.82%) and 523 girls (78.18%). There were 338 freshmen (50.52%), 245 sophomores (36.62%), 35 juniors (5.23%), and 51 seniors (7.63%); there were 169 urban college students (25.26%) and 500 rural college students (74.74%). There were 122 only children (18.24%) and 547 non-only children (81.76%).

Prior to participation, students were informed that their involvement was voluntary and that all responses would remain anonymous. Written informed consent was obtained from all participants, and parents of the research participants under the age of 18 provided informed consent. The research procedure has been approved by the Academic Ethics Committee of College of Education, Hunan University of Science and Technology.

### 3.2 Measures

#### 3.2.1 Psychologically rich life questionnaire

The Psychologically Rich Life Questionnaire (Oishi et al., 2019) was used, which was developed by Oishi and revised by (Wang et al. 2022b). The questionnaire consists of 12 items, response options ranged from 1 (strongly disagree) to 7 (strongly agree), and the average score for all items was calculated to measure participants' psychological richness. A higher score indicates a richer psychology. The Cronbach's α coefficient of the questionnaire in this study was 0.92. The results of confirmatory factor analysis showed that the scale model fitted well: χ^2^/df = 4.319, *p* < 0.001, RMSEA = 0.070, CFI = 0.963, TLI = 0.952, SRMR = 0.029. This shows that the scale has good reliability and validity (Wen et al., 2004; Cicchetti, 1994; Hu and Bentler, 1999), can effectively measure the psychological richness of Chinese college students.

#### 3.2.2 Cognitive flexibility scale

The Cognitive Flexibility Scale was developed by (Martin and Rubin 1995) and revised by (Qi et al. 2012). The scale consists of 13 items, of which 2, 3, 6, and 10 questions are scored in reverse. A 6-point rating scale was used, with 1 indicating “very disagreeable” and 6 indicating “very agreeable.” A higher score indicates that the participants have a higher level of cognitive flexibility. In the present study, the Cronbach's α coefficient of the scale was 0.80. The results of confirmatory factor analysis showed that the scale model fitted well: χ^2^/df = 2.401, *p* < 0.001, RMSEA = 0.046, CFI = 0.968, TLI = 0.954, SRMR = 0.030. This shows that the scale has good reliability and validity (Wen et al., 2004; Cicchetti, 1994; Hu and Bentler, 1999), can effectively measure the cognitive flexibility of Chinese college students.

#### 3.2.3 Creative self-efficacy scale

This study utilized the Creative Self-Efficacy Scale, which was developed by (Carmeli and Schaubroeck 2007) and was revised by (Patrick 2019). The scale consists of 8 items, rated on a 5-point scale (1–5), ranging from 1 (strongly disagree) to 5 (strongly agree), with the higher average score for all items indicating higher creative self-efficacy. In this study, the Cronbach's α coefficient of this scale was 0.91. The results of confirmatory factor analysis showed that the scale model fitted well: χ^2^/df = 4.342, *p* < 0.001, RMSEA = 0.071, CFI = 0.980, TLI = 0.969, SRMR = 0.024. This shows that the scale has good reliability and validity (Wen et al., 2004; Cicchetti, 1994; Hu and Bentler, 1999), can effectively measure the creative self-efficacy of Chinese college students.

#### 3.2.4 Innovative Behavior Scale

The Innovative Behavior Scale was developed by (Scott and Bruce 1994) and revised by (Xin 2016), which included 5 items. The same five-point Likert scale was used, ranging from 1 (strongly disagree) to 5 (strongly agree), with the higher average score for all items indicating higher innovative behavior. This study uses self-reports to measure the innovative behavior of college students. In this study, the Cronbach's α coefficient of the scale is 0.80. The results of confirmatory factor analysis showed that the scale model fitted well: χ^2^/df = 2.566, *p* < 0.001, RMSEA = 0.048, CFI = 0.995, TLI = 0.985, SRMR = 0.015. This shows that the scale has good reliability and validity (Wen et al., 2004; Cicchetti, 1994; Hu and Bentler, 1999), can effectively measure the innovative behavior of Chinese college students.

### 3.3 Statistical analysis

SPSS 25.0 and Mplus8.3 were used to analyze the data. SPSS 25.0 was used for common method bias, descriptive statistics, Kolmogorov-Smirnov normality test (K-S test), and correlation analysis of the data. Mplus8.3 was used to model the structural equation model (SEM) and verify the chain mediation effect. First, the Kolmogorov-Smirnov test was used to test the normality distribution of the data, and the results showed that the scores of the study participants on the four scales did not obey the normal distribution (*p* < 0.05), and all four scales were unidimensional. If the original questions are used to construct the equation model will produce serious parameter estimation bias (Wu and Wen, 2011). Combined with the suggestion of (Wu and Wen 2011), this study used the item packing strategy to construct the structural equation model so as to reduce the measurement error and improve the overall model fit. Second, the results of the validation factor analysis showed that the models of the unidimensional structure of the four scales were well-fitted and the factor loadings of all the items were above 0.4, with a high degree of homogeneity of the entries within the scales, which suggests that each of the scales satisfies the single-dimensionality and homogeneity, and applies to the item packaging strategy. Meanwhile, the chain mediation model constructed using the item packing strategy has a good fit index [χ^2^/df = 2.33, *p* < 0.001, RMSEA = 0.05, CFI = 0.99, TLI = 0.98, SRMR = 0.02], which is significantly better than that of the model before packing grouping [χ^2^/df = 2.75, *p* < 0.001, RMSEA = 0.05, CFI = 0.87, TLI = 0.86, SRMR = 0.05], a result that also suggests the necessity of using an item packing strategy.

To minimize the potential biases and errors associated with this method, we took the following steps: first, packaged and grouped items only within scales to avoid mispackaging; second, used a factorial-balanced method to package and group items, which improves more stable parameter estimates; and third, each unidimensional scale was grouped into two or three indicators (all items of psychological richness and cognitive flexibility were grouped into three items each), and all items of creative self-efficacy and innovative behaviors were grouped into two items each, which helps to improve the fit of the model and to control parameter estimation bias.

## 4 Results

### 4.1 Common method bias tests

In the present study, Harman's single-factor test was used to examine the common method bias (Zhou and Long, 2004). All the items in the four variables of psychological richness, cognitive flexibility, creative self-efficacy, and innovative behavior underwent exploratory factor analysis (unrotated). The results identified 6 factors with eigenvalues >1. Importantly, the first factor explained 35.91% of the total variance, which is below the critical threshold of 40% (Tang and Wen, 2020). Therefore, there is no significant common method bias in this study.

### 4.2 Descriptive statistics and correlation analysis

Descriptive statistics and correlation analysis of each variable are presented in Table 1. The results indicate that psychological richness is positively correlated with cognitive flexibility (*p* < 0.01), creative self-efficacy (*p* < 0.01), and innovative behavior (*p* < 0.01). Moreover, cognitive flexibility is positively correlated with creative self-efficacy and innovative behavior (*p* < 0.01), respectively. Additionally, creative self-efficacy is significantly positively correlated with innovative behavior (*p* < 0.01). However, gender and age were not significantly correlated with the above four variables (*p* > 0.05).

**Table 1:** Descriptive statistics and correlation analysis results.

	**M**	**SD**	**1**	**2**	**3**	**4**	**5**	**6**
1 Gender (1 = boy, 2 = girl)	1.78	0.41	1					
2 Age	19.18	1.50	0.01	1				
3 Psychological richness	4.79	1.03	−0.03	−0.06	1			
4 Cognitive flexibility	51.01	6.83	−0.02	−0.04	0.56^**^	1		
5 Creative self-efficacy	3.41	0.67	−0.07	−0.02	0.61^**^	0.66^**^	1	
6 Innovative behavior	3.24	0.58	−0.02	−0.02	0.59^**^	0.64^**^	0.73^**^	1

^**^Represent *p* < 0.01.

### 4.3 The chain mediating effect analysis

**Figure 2:**
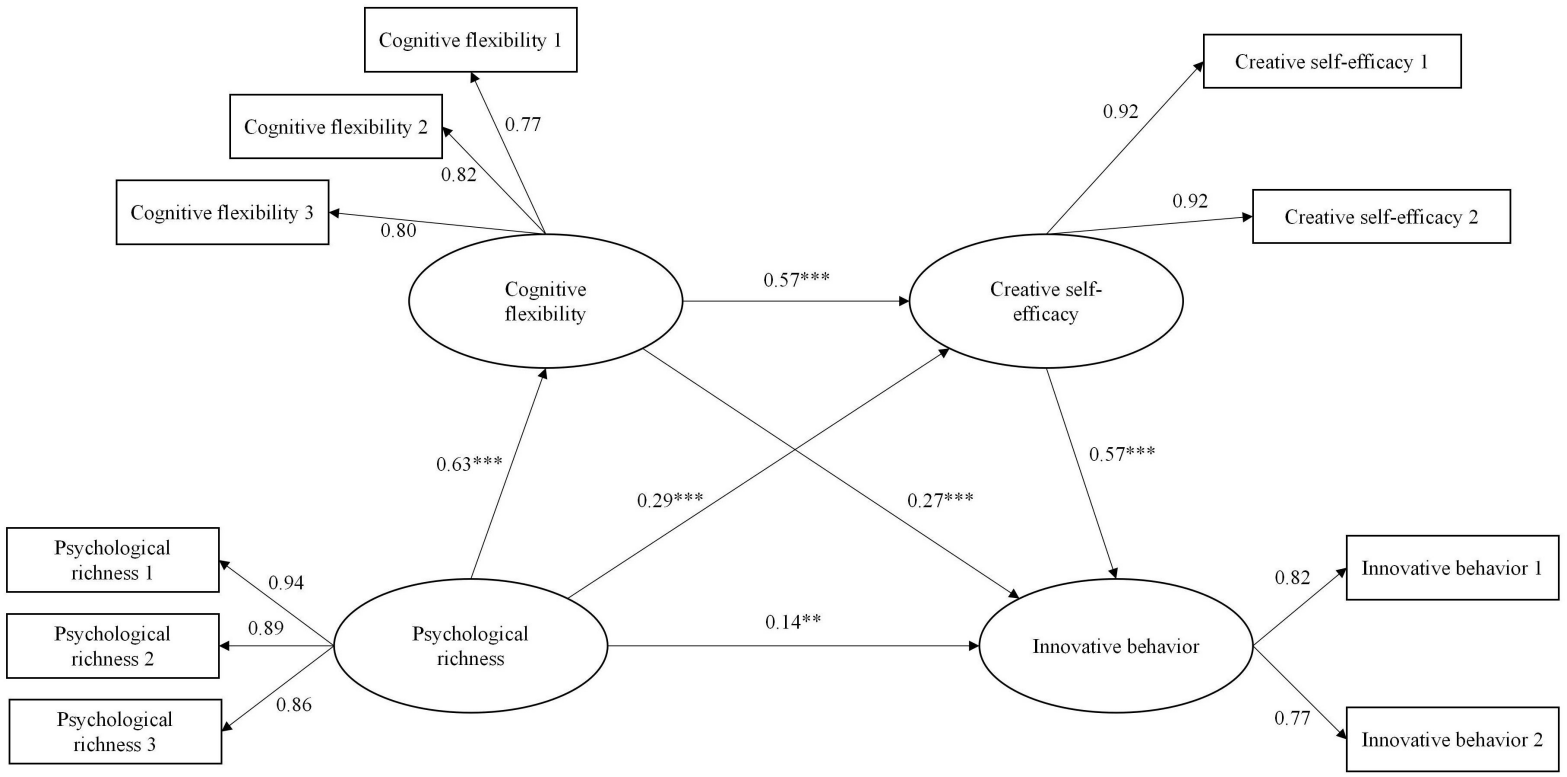
The path diagram of chain mediation between cognitive flexibility and creative self-efficacy. **p < 0.01, ***p < 0.001.

Given the influence of gender and age on the innovative behavior of college students (Mei et al., 2015; Stevenson et al., 2014). In this study, gender and age were treated as control variables, and the utilization of the latent variable structural equation model was used to test the mediating effects of cognitive flexibility and creative self-efficacy on the relationship between psychological richness and innovative behavior of college students. The results showed that the chain mediation model had a good fitting index [χ^2^/df = 2.33, *p* < 0.001, RMSEA = 0.05, CFI = 0.99, TLI = 0.98, SRMR = 0.02]. As illustrated in Figure 2, all path coefficients within the model reached statistical significance. Notably, the structural model revealed significant direct relationships among the four variables. Specifically, psychological richness positively predicts cognitive flexibility (β = 0.63, *p* < 0.001), creative self-efficacy (β = 0.29, *p* < 0.001), and innovative behavior (β = 0.14, *p* = 0.004). Furthermore, cognitive flexibility positively predicts creative self-efficacy (β = 0.57, *p* < 0.001) and innovative behavior (β = 0.27, *p* < 0.001). In addition, Creative self-efficacy positively predicts innovative behavior (β = 0.57, *p* < 0.001). The bias-corrected percentile Bootstrap method (repeated sampling 5,000 times) was used to test the mediating effect. The results showed that the 95% confidence intervals for all pathways did not include 0, indicating that the separate mediating effect and chain mediating effect of cognitive flexibility and creative self-efficacy were significant. Hypotheses 1–4 were supported. The specific mediating effect values and confidence intervals are shown in Table 2. Furthermore, the predictive effects of gender and age on innovative behavior were not significant (*p* > 0.05).

**Table 2:** Effect values and confidence intervals of Bootstrap path coefficients.

**Paths**	**Effect**	**BootSE**	**BootLLCI**	**BootULCI**	**Percentage (%)**
P → RIB	0.14	0.05	0.04	0.23	20.59%
PR → CF → IB	0.17	0.04	0.09	0.26	25.00%
PR → CSE → IB	0.17	0.03	0.11	0.24	25.00%
PR → CF → CSE → IB	0.20	0.03	0.15	0.27	29.41%
Total effect	0.68	0.03	0.62	0.74	

PR, psychological richness; CF, cognitive flexibility; CSE, creative self-efficacy; IB, innovative behavior.

## 5 Discussion

This study investigated the influence of psychological richness on innovative behavior among Chinese college students and examined the underlying mechanism by constructing a chain mediation model. The findings suggest that psychological richness can affect innovative behavior through the mediating effects of cognitive flexibility and creative self-efficacy, as well as the chain mediating effect of the two.

### 5.1 The relationship between psychological richness and innovative behavior among Chinese college students

The findings of this study indicate a significant positive correlation between psychological richness and the Chinese innovative behavior of college students, confirming H1. This study replicated previous research findings (Xu, 2025), supporting the idea that psychological richness can positively predict the innovative behavior of college students. It further corroborates the social cognitive theory. Rich life experiences not only change the emotional state and cognitive perspective of college students but also promote the development of agency (Oishi and Westgate, 2022; Oishi et al., 2019). Agency is the key driving force for the development of college students' innovative behavior (Luo et al., 2025). When this driving force is reinforced, college students are more motivated and more actively pursue the satisfaction of cognitive needs, and more actively participate in novel and difficult activities, thus showing more innovative behavior (Besser and Oishi, 2020; Selznick et al., 2022). At the same time, psychological richness is closely related to openness. College students with psychological richness often have an open personality (Oishi et al., 2019), which makes them more receptive to new ideas and new things, more imaginative, and more likely to participate in various innovative behaviors (Adams et al., 2025). In addition, rich life experience can promote the development of college students' social skills (Gu et al., 2023). College students who actively participate in social interactions can be exposed to diverse ideas and concepts. This provides direct material for the generation of innovative ideas and thus promotes the development of innovative behavior.

### 5.2 The mediating role of cognitive flexibility

This study found that psychological richness can affect Chinese college students' innovative behavior through the separate mediation of cognitive flexibility, confirming H2. This finding is consistent with previous research findings (Xu et al., 2019), that cognitive flexibility can mediate the impact of psychological traits on college students' innovative activities. Firstly, psychological richness comes from diverse life experiences. According to constructivist theory, diverse and complex life experiences can expand the individual's perspective on problems (Oishi et al., 2024), thereby reconstructing the original cognitive framework and thus improving the flexibility of thinking (Oishi et al., 2021). Studies have found that psychologically rich college students are more interested in short-distance travel (Oishi et al., 2021). They can encounter new things on the journey, feel positive emotions, deepen their understanding of the diversity and complexity of the world, and then significantly improve their cognitive flexibility (De Bloom et al., 2014). Meanwhile, it is also a validation of constructivist theory in the field of positive psychology. Secondly, cognitive flexibility is a core element of innovative ability (Amabile, 1988); the innovative behavior of college students often involves cognitive activities. Flexible cognitive ability can help individuals break rigid thinking patterns, cope with challenges by integrating knowledge from different fields, and thus promote the development of innovative behavior.

### 5.3 The mediating role of creative self-efficacy

This study found that creative self-efficacy indirectly links psychological richness to innovative behavior of Chinese college students, confirming H3. Our findings support the theory of self-efficacy (Bandura, 1977), which suggests that psychological richness has a positive impact on innovative behavior by enhancing creative self-efficacy. The psychologically rich life is full of difficulties and challenges (Oishi and Westgate, 2022). In the process of overcoming difficulties and coping with challenges, individuals will continuously improve their ability to adapt to uncertain events and solve problems encountered in life with their abilities. This will strengthen their confidence in participating in novel activities, thereby enhancing their sense of creative self-efficacy. Meanwhile, creative self-efficacy is a key prerequisite for the realization of innovative behaviors (Yang et al., 2011). On the one hand, it can stimulate individuals' interest in participating in innovative activities and deepen their curiosity about novel things and unknown fields, thereby promoting the emergence of innovative behaviors (Wang et al., 2016). On the other hand, it can effectively improve the individuals' self-esteem levels (Liao et al., 2023), and college students with higher self-esteem perform better in innovative behaviors (Chen et al., 2022).

### 5.4 The chain-mediated effect of cognitive flexibility and creative self-efficacy

This study further reveals the chain-mediated effect of cognitive flexibility and creative self-efficacy on the relationship between psychological richness and innovative behavior of Chinese college students. H4 has been confirmed. Specifically indicating that psychological richness enhances college students' cognitive flexibility, thereby enhancing their creative self-efficacy and promoting innovative behavior. According to the social cognitive theoretical framework and constructivist theory, the process of psychological richness is the process of meaning construction. With the accumulation of diversified experiences, the cognitive schema will become more complicated. Rich cognitive schema can help individuals solve problems from multiple angles when dealing with challenges, thus improving their cognitive flexibility. Individuals with high cognitive flexibility tend to favor controllability attribution, believing that the difficulties encountered can be solved entirely through their efforts and abilities (Dennis and Vander Wal, 2010). This style of attribution can help them to significantly increase their creative self-efficacy when they are completing a challenging task. Meanwhile, college students with high creative self-efficacy are more likely to actively engage in creative conceptualization, proactively explore new knowledge and technologies to realize their ideas, and exhibit more innovative behaviors. In addition, in this study, gender and age, as control variables, had no significant effect on psychological richness, cognitive flexibility, innovative self-efficacy, and innovative behavior. This may reflect the relative homogeneity of the gender and age distribution of the samples in this study. For example, the samples are mainly female students, and most of them are lower grade students, and the age is concentrated in the 18–20 years old, which limits the scope of the significant role of these two demographic variables.

### 5.5 Implications, limitations, and future direction

The present research reveals, for the first time, the relationship between psychological richness and Chinese college students' innovative behaviors and the intrinsic mechanism of action, which enriches the results of positive psychology in the field of higher education in China and is conducive to the promotion and application of positive psychology in the context of Chinese culture. At the same time, this research validates the path of “personal factors affecting behavioral development” and further expands the application scope of social cognitive theory, which not only promotes the development of social cognitive theory but also provides theoretical support for the cultivation of innovative behaviors among Chinese college students. At the practical level, this study explores the process of psychological richness on college students' innovative behaviors in the context of Chinese culture, which is insightful for the cultivation of innovative talents in Chinese higher education: firstly, universities can design courses based on the perspective of psychological richness, and set up cross-disciplinary contents in the courses, such as “Artificial Intelligence and Art Design,” “Biotechnology and Philosophical contemplation” and other integrated courses, so that students can accumulate diversified experiences in solving practical problems (e.g., environmental protection projects). Secondly, educators should focus on the cultivation of students' curiosity and build teaching situations that contribute to emotional experience and perspective change, such as “flipped classroom” and “knowledge competition” activities, to guide students to cope with challenges and overcome difficulties, so as to achieve psychological richness. Thirdly, universities can establish a diversified student evaluation system, in addition to the traditional evaluation of students' grades, they should also enhance the evaluation of college students' experience and gains in innovation practice, for example, encouraging college students to participate in scientific research, entrepreneurship and other activities, which will stimulate college students' cognitive flexibility and creative self-efficacy.

This study also has some limitations. First, the variables in this study were measured by a single self-report, which may be subject to response bias, and subsequent studies could combine behavioral and physiological indicators to jointly assess these four variables. Secondly, although this study constructs a chain mediation model through a cross-sectional survey, it cannot test the causal relationship. Future studies could employ longitudinal or experimental designs to further verify the relationship between variables. Thirdly, this study focuses on the effects of individual psychological factors on innovative behavior, but does not include external contextual variables such as organizational support and educational policies. Future research can expand the multilevel analysis framework to explore the interaction of external contextual factors (e.g., teachers' teaching styles, allocation of innovation resources) and individual factors on the synergistic effect on innovative behavior. Fourth, the data in this study came from only three colleges and universities in Hunan and Shaanxi provinces, and the sample in this study had a high proportion of female students and was mainly concentrated in the freshman and sophomore grades; moreover, this study ignored the influence of academic background on the study variables, which may limit the generalizability of the findings to a wider population. Future research may employ stratified sampling techniques to gather data from colleges and universities across various regions. This approach should appropriately increase the representation of male students and senior students within the sample, while also taking into account the impact of academic background. Such measures will enhance the generalizability and external validity of the findings. Fifth, the item parceling strategy of scale items may mask important latent variables, thereby affecting the reliability of the research results. Subsequent research can conduct an in-depth analysis and discussion on the error correlation of the questions before packaging and grouping, and combine the latent category analysis method to test whether there are important latent variables, thereby enhancing the validity of the research results. Sixth, this study mainly focuses on college students in the context of Chinese culture and fails to fully consider the influence of cultural differences on the research results. To some extent, this limits the universal applicability of the research conclusions in other cultural contexts. Future research can conduct cross-cultural comparative studies, collect sample data from different cultural environments, deeply explore the influence of cultural factors on research results, and further enhance the generalizability of the research.

## 6 Conclusion

The main research conclusions of this study are as follows: (1) Psychological richness has a significant positive predictive effect on college students' innovative behavior; (2) Cognitive flexibility and creative self-efficacy have a significant independent mediating effect. Psychological richness can affect college students' innovative behavior through cognitive flexibility or creative self-efficacy; (3) The chain mediating effect between cognitive flexibility and creative self-efficacy is significant, and psychological richness can enhance creative self-efficacy by enhancing cognitive flexibility, thereby increasing innovative behavior.

## Data Availability

The original contributions presented in the study are included in the article/supplementary material, further inquiries can be directed to the corresponding author.

## References

[B1] Aboobaker N. Zakkariya K. A. (2019). Influence of digital learning orientation and readiness for change on innovative work behaviour: reflections from the higher education sector. Dev. Learn. Organ. 34, 25–28. 10.1108/DLO-08-2019-0191

[B2] Adams D. Bellibaş M. S. Muniandy V. Pietsch M. (2025). The role of openness to experience in innovating teaching and instruction through leader-member exchange and teacher creativity. Think Skills Creat. 57:101834. 10.1016/j.tsc.2025.101834

[B3] Afsar B. Masood M. (2018). Transformational leadership, creative self-efficacy, trust in supervisor, uncertainty avoidance, and innovative work behavior of nurses. J. Appl. Behav. Sci. 54, 36–61. 10.1177/0021886317711891

[B4] Agaoglu F. O. Bas M. Tarsuslu S. Ekinci L. O. (2025). Serial mediating role of transformational leadership and perception of artificial intelligence use in the effect of employee happiness on innovative work behaviour in nurses. BMC Nurs. 24:137. 10.1186/s12912-025-02776-9 39915749 PMC11800594

[B5] Al-Mamary Y. H. Alshallaqi M. (2022). Impact of autonomy, innovativeness, risk-taking, proactiveness, and competitive aggressiveness on students' intention to start a new venture. J. Innov. Knowl. 7:100239. 10.1016/j.jik.2022.100239

[B6] Alt D. Kapshuk Y. Dekel H. (2023). Promoting perceived creativity and innovative behavior: benefits of future problem-solving programs for higher education students. Think Skills Creat. 47:101201. 10.1016/j.tsc.2022.101201

[B7] Amabile T. M. (1988). A model of creativity and innovation in organizations. Res. Organ. Behav. 10, 123–167.

[B8] Bandura A. (1977). Self-efficacy: toward a unifying theory of behavioral change. Psychol. Rev. 84:191. 10.1037/0033-295X.84.2.191 847061

[B9] Bandura A. (1986). Social Foundations of Thought and Action: A Social Cognitive Theory. Engle-wood Cliffs, NJ: Prentice Hall.

[B10] Bandura A. (1989). Human agency in social cognitive theory. Am. Psychol. 44:1175. 10.1037/0003-066X.44.9.1175 2782727

[B11] Besser L. L. Oishi S. (2020). The psychologically rich life. Philos. Psychol. 33, 1053–1071. 10.1080/09515089.2020.1778662

[B12] Büning C. Jürgens L. Lausberg H. (2021). Divergent learning experiences in sports enhance cognitive executive functions and creativity in students. Phys Educ Sport Pedagogy 6, 402–416. 10.1080/17408989.2020.1812056

[B13] Carmeli A. Schaubroeck J. (2007). The influence of leaders' and other referents' normative expectations on individual involvement in creative work. Leadersh Q. 18, 35–48. 10.1016/j.leaqua.2006.11.001

[B14] Central Committee of the Communist Party of China and State Council (2019). Available online at: https://www.gov.cn/zhengce/2019-02/23/content_5367987.htm (Accessed Februar*y* 23, 2019).

[B15] Chen X. He J. Fan X. (2022). Relationships between openness to experience, cognitive flexibility, self-esteem, and creativity among bilingual college students in the U.S. Int. J. Biling. Educ. Biling. 25, 342–354. 10.1080/13670050.2019.1688247

[B16] Chin D. Kameoka V. A. (2002). Psychosocial and Contextual Predictors of Educational and Occupational Self-Efficacy among Hispanic Inner-City Adolescents. Hisp. J. Behav. Sci. 24, 448–464. 10.1177/0739986302238214

[B17] Cicchetti D. V. (1994). Guidelines, criteria, and rules of thumb for evaluating normed and standardized assessment instruments in psychology. Psychol Assess. 6:284. 10.1037/1040-3590.6.4.284

[B18] De Bloom J. Ritter S. Kühnel J. Reinders J. Geurts S. (2014). Vacation from work: a ‘ticket to creativity'? Tour Manag. 44, 164–171. 10.1016/j.tourman.2014.03.013

[B19] De Dreu C. K. W. Baas M. Nijstad B. A. (2008). Hedonic tone and activation level in the mood-creativity link: toward a dual pathway to creativity model. J. Pers. Soc. Psychol. 94, 739–756. 10.1037/0022-3514.94.5.739 18444736

[B20] Dennis J. P. Vander Wal J. S. (2010). The cognitive flexibility inventory: instrument development and estimates of reliability and validity. Cognit. Ther. Res. 34, 241–253. 10.1007/s10608-009-9276-427409075

[B21] Diener E. Lucas R. E. Oishi S. (2018). Advances and open questions in the science of subjective well-being. Collabra Psychol. 4:15. 10.1525/collabra.115 30637366 PMC6329388

[B22] Dreu C. K. W. D. Nijstad B. A. Baas M. (2011). Behavioral activation links to creativity because of increased cognitive flexibility. Soc. Psychol. Pers. Sci. 2, 72–80. 10.1177/1948550610381789

[B23] Ford C. M. (1996). A theory of individual creative action in multiple social domains. Acad. Manag. Rev. 21, 1112–1142. 10.2307/25916629312046

[B24] Gökçe S. Güner P. (2024). Pathways from cognitive flexibility to academic achievement: mediating roles of critical thinking disposition and mathematics anxiety. Curr. Psychol. 43, 18192–18206. 10.1007/s12144-024-05642-0

[B25] Gu Y. Tao L. Zheng W. (2023). Relationship between COVID-19-related stress and social inhibition among university students in China: the mediating role of psychological richness. Soc. Pers. Psychol. Compass. 17:e12872. 10.1111/spc3.12872

[B26] Guo J. Deng F. (2020). On the influence of the talent cultivation reform on the innovation ability of college students. J. High. Educ. 41, 70–77.

[B27] Hu L. Bentler P. M. (1999). Cutoff criteria for fit indexes in covariance structure analysis: conventional criteria versus new alternatives. Struct. Equ. Model. 6, 1–55. 10.1080/1070551990954011836787513

[B28] Huang W. Zhang S. (2024). The impact of meaning in life on preservice teachers' innovative behavior: the chain mediating effects of career calling and learning engagement. Curr. Psychol. 43, 18294–18306. 10.1007/s12144-024-05671-9

[B29] Iqbal A. Nazir T. Ahmad M. S. (2022). Entrepreneurial leadership and employee innovative behavior: an examination through multiple theoretical lenses. Eur. J. Innov. Manag. 25, 173–190. 10.1108/EJIM-06-2020-0212

[B30] Keyser V. Barling J. (1981). Determinants of children's self-efficacy beliefs in an academic environment. Cognit. Ther. Res. 5, 29–39. 10.1007/BF01172324

[B31] Kurtovic A. Vrdoljak G. Idzanovic A. (2019). Predicting procrastination: the role of academic achievement, self-efficacy and perfectionism. Int. J. Educ. Psychol. 8:1. 10.17583/ijep.2019.2993

[B32] Kwon S.-H. Kim J.-S. (2025). Relationship between participative decision-making within an organization and employees' cognitive flexibility, creativity, and voice behavior. Behav. Sci. 15:51. 10.3390/bs15010051 39851854 PMC11762380

[B33] Li J. B. Dou K. Liang Y. (2021). The relationship between presence of meaning, search for meaning, and subjective well-being: a three-level meta-analysis based on the meaning in life questionnaire. J. Happiness Stud. 22, 467–489. 10.1007/s10902-020-00230-y

[B34] Li X. Zhang B. Jiang L. (2019). An empirical study of the factors of college students' innovative behavior. Educ. Res. 40, 91–100.

[B35] Liao J. Xia T. Xu X. Pan L. (2023). The effect of appearance anxiety on social anxiety among college students: sequential mediating effects of self-efficacy and self-esteem. Behav. Sci. 13:692. 10.3390/bs13080692 37622832 PMC10451712

[B36] Lin M.-Y. Chang Y.-S. (2024). Using design thinking hands-on learning to improve artificial intelligence application creativity: a study of brainwaves. Think Skills Creat. 54:101655. 10.1016/j.tsc.2024.101655

[B37] Liu D. Wu C. Meng Y. Dang J. (2025). How self-belief in creativity and well-being is associated with life satisfaction, meaning in life, and psychological richness: the mediating effect of creative self-efficacy. J. Intell. 13:25. 10.3390/jintelligence13030025 40137057 PMC11942731

[B38] Liu R. Jong C. Fan M. (2024). Reciprocal relationship between self-efficacy and achievement in mathematics among high school students: First author. Large Scale Assess. Educ. 12:14. 10.1186/s40536-024-00201-236211864

[B39] Luo L. Hu J. Zheng Y. Li C. (2025). Human vs. AI: does AI learning assistant enhance students' innovation behavior? *Educ. Inf. Technol*. 1–48. 10.1007/s10639-025-13474-z

[B40] Ma K. Zhang Y. Hui B. (2024). How does AI affect college? The impact of AI usage in college teaching on students' innovative behavior and well-being. Behav. Sci. 14:1223. 10.3390/bs14121223 39767364 PMC11673235

[B41] Martin M. M. Rubin R. B. (1995). A new measure of cognitive flexibility. Psychol. Rep. 76, 623–626. 10.2466/pr0.1995.76.2.623

[B42] Mei H. Ren Z. Feng G. Yang S. Hu S. (2015).Does innovation support change college students' innovative behavior? Fud. Educ. Forum. 13, 26–32. 10.13397/j.cnki.fef.2015.06.005

[B43] Mishra A. Singh P. (2024). Effect of emotional intelligence and cognitive flexibility on entrepreneurial intention: mediating role of entrepreneurial self-efficacy. J. Entrep. Emerg. Econ. 16, 551–575. 10.1108/JEEE-05-2022-0142

[B44] Mortimer J. A. Ding D. Borenstein A. R. DeCarli C. Guo Q. Wu Y.. (2012). Changes in brain volume and cognition in a randomized trial of exercise and social interaction in a community-based sample of non-demented Chinese elders. J. Alzheimers Dis. 30, 757–766. 10.3233/JAD-2012-120079 22451320 PMC3788823

[B45] Oishi S. Choi H. Buttrick N. Heintzelman S. J. Kushlev K. Westgate E. C.. (2019). The psychologically rich life questionnaire. J. Res. Pers. 81, 257–270. 10.1016/j.jrp.2019.06.010

[B46] Oishi S. Choi H. Liu A. Kurtz J. (2021). Experiences associated with psychological richness. Eur. J. Pers. 35, 754–770. 10.1177/0890207020962334

[B47] Oishi S. Westgate E. Cha Y. (2024). The cognitive complexity of a happy life, a meaningful life, and a psychologically rich life. J. Res. Pers. 110:104475. 10.1016/j.jrp.2024.104475

[B48] Oishi S. Westgate E. C. (2022). A psychologically rich life: beyond happiness and meaning. Psychol. Rev. 129, 790–811. 10.1037/rev0000317 34383524

[B49] Oishi S. Westgate E. C. (2025). Psychological richness offers a third path to a good life. Trends Cogn. Sci. Adv. 10.1016/j.tics.2025.04.002 40280834

[B50] Orakci S. Khalili T. (2025). The impact of cognitive flexibility on prospective EFL teachers' critical thinking disposition: the mediating role of self-efficacy. Cogn. Process. 26, 59–73. 10.1007/s10339-024-01227-8 39215787

[B51] Pajares F. Graham L. (1999). Self-efficacy, motivation constructs, and mathematics performance of entering middle school students. Contemp. Educ. Psychol. 24, 124–139. 10.1006/ceps.1998.0991 10072312

[B52] Patrick K. (2019). The Influence of Chinese and German College Students' Innovative Self-efficacy on Entrepreneurial Behavior—the Mediating Role of Entrepreneurial Intention (Master's Thesis). Xiamen University, Xiamen, China.

[B53] Qi B. Zhao B. Wang K. Liu H. (2012). Revision and preliminary application of cognitive flexibility scale for college students. Stud. Psychol. Beha. 11, 120–123.27409075

[B54] Raskin J. D. (2011). On essences in constructivist psychology. J. Theor. Philos. Psychol. 31, 223–239. 10.1037/a0025006

[B55] Ruscio A. M. Amabile T. M. (1999). Effects of instructional style on problem-solving creativity. Creat. Res. J. 12, 251–266. 10.1207/s15326934crj1204_3

[B56] Ryan R. M. Deci E. L. (2000). Self-determination theory and the facilitation of intrinsic motivation, social development, and well-being. Am. Psychol. 55:68. 10.1037/0003-066X.55.1.68 11392867

[B57] Ryff C. D. (1989). Happiness is everything, or is it? Explorations on the meaning of psychological well-being. J. Pers. Soc. Psychol. 57:1069. 10.1037/0022-3514.57.6.1069

[B58] Salam S. Senin A. A. (2022). A bibliometric study on innovative behavior literature (1961–2019). Sage Open 12:21582440221109589. 10.1177/21582440221109589

[B59] Scott S. G. Bruce R. A. (1994). Determinants of innovative behavior: a path model of individual innovation in the workplace. Acad. Manage. J. 37, 580–607. 10.2307/256701

[B60] Selznick B. S. Dahl L. S. Youngerman E. Mayhew M. J. (2021). Equitably linking integrative learning and students' innovation capacities. Innov. High. Educ. 47, 1–21. 10.1007/s10755-021-09570-w 34334931 PMC8308063

[B61] Selznick B. S. Mayhew M. J. Zhang L. McChesney E. T. (2022). Building women's innovation capacities through undergraduate experiences. Res. High. Educ. 63, 567–588. 10.1007/s11162-021-09659-3

[B62] Stevenson C. E. Kleibeuker S. W. De Dreu C. K. W. Crone E. A. (2014). Training creative cognition: adolescence as a flexible period for improving creativity. Front. Hum. Neurosci. 8:827. 10.3389/fnhum.2014.00827 25400565 PMC4212808

[B63] Tang D. Wen Z. (2020). Statistical approaches for testing common method bias: problems and suggestions. J. Psychol. Sci. 43, 215–223. 10.16719/j.cnki.1671-6981.20200130

[B64] Tierney P. Farmer S. M. (2002). Creative self-efficacy: its potential antecedents and relationship to creative performance. Acad. Manag. J. 45, 1137–1148. 10.2307/306942938098535

[B65] Wang N. Zhang L. Wang Y. (2016). Influence of creative self-efficacy on innovative behavior: analysis of multiple mediation effect. Stud. Psychol. Beha. 14, 811–816.

[B66] Wang X. (2005). Three Transformations in study theories driven by constructivism. Psychol. Sci. 6, 242–244. 10.16719/j.cnki.1671-6981.2005.06.06232027574

[B67] Wang X.-M. Huang X.-T. Han Y.-H. Hu Q.-N. (2024). Promoting students' creative self-efficacy, critical thinking and learning performance: an online interactive peer assessment approach guided by constructivist theory in maker activities. Think Skills Creat. 52:101548. 10.1016/j.tsc.2024.101548

[B68] Wang Y. Huang S. Chen C. Liu X. (2022a). Influence of psychological capital on college students' innovative behavior: juxtaposing mediating effect of intrinsic motivation. Chin. J. Health Psychol. 30, 422–426. 10.13342/j.cnki.cjhp.2022.03.021

[B69] Wang Y. Ren X. Zhang L. (2022b). Reliability and validity of the psychologically rich life questionnaire in college students. Chin. J. Clin. Psychol. 30, 936–939. 10.16128/j.cnki.1005-3611.2022.04.03527409075

[B70] Wei X. Yu F. (2024). The concept, measurement, function and future directions of psychological richness. Psychol. Res. 17, 483–490. 10.19988/j.cnki.issn.2095-1159.2024.06.001

[B71] Wen Z. Hau K.-T. Marsh H. W. (2004). Structural equation model testing: cutoff criteria for goodness of fit indices and chi-square test. Acta Psychol. Sin. 36, 186–194.

[B72] Wu Y. Wen Z. (2011). Item parceling strategies in structural equation modeling. Adv. Psychol. Sci. 19, 1859–1867. 10.3724/SP.J.1042.2011.0185937113526

[B73] Xin T. (2016). Research on Affecting Factors of College Students' Innovation Behaviors (Master's Thesis). Harbin Institute of Technology, Harbin, China.

[B74] Xu W. Yang G. Zhu M. Chen J. Li W. (2019). Effect of psychological resilience on creativity: the mediating role of cognitive flexibility. Chin. J. Health Psychol. 27, 1885–1890. 10.13342/j.cnki.cjhp.2019.12.034

[B75] Xu X. (2025). The impact of psychological richness on creativity among college students under the ecological psychological perspective and intervention strategies. Psychol. Mont. 20, 136–138. 10.19738/j.cnki.psy.2025.07.037

[B76] Yang J. Yang D. Zhao S. Jing L. (2011). The motivation of employee creativity: employee creative self-efficacy. Adv. Psychol. Sci. 19, 1363–1370. 10.3724/SP.J.1042.2011.0136337113526

[B77] Yang Y. Wu S. Duñabeitia J. A. Jiang K. Li Y. (2021). The influence of L2 proficiency on bilinguals' creativity: the key role of adaptive emotion regulation strategies during the COVID-19 pandemic. Front. Psychol. 12:695014. 10.3389/fpsyg.2021.695014 34539492 PMC8442584

[B78] Yu F.-Y. Su C.-L. (2015). A student-constructed test learning system: the design, development and evaluation of its pedagogical potential. Australas. J. Educ. Technol. 31, 685–698. 10.14742/ajet.2190

[B79] Yu G. Hou R. (2004). Problem raising, cognitive styles and the cultivation of creativity in school education. Educ. Sci. 20, 54–58.

[B80] Yu G. Zhang W. (2019). Creativity and mental health: an interpretation from a relational perspective. J. Chin. Socie. Educ. 13–18.

[B81] Yu X. Zhao X. Hou Y. (2023). Cognitive flexibility and entrepreneurial creativity: the chain mediating effect of entrepreneurial alertness and entrepreneurial self-efficacy. Front. Psychol. 14:1292797. 10.3389/fpsyg.2023.1292797 38098535 PMC10720366

[B82] Zheng Y. (2023). The influence mechanism of learning motivation on college students' innovative behavior: mediating effect of extracurricular activities. Fud. Educ. Forum 21, 63–71. 10.13397/j.cnki.fef.2023.05.008

[B83] Zhou H. Long L. (2004). Statistical remedies for common method biases. Adv. Psychol. Sci. 12, 942–950.

[B84] Zhou M. Yu K. Wang F. (2021). Cognitive flexibility and individual adaptability: a cross-lag bidirectional mediation model. Chin. J. Clin. Psychol. 29, 182–186+190. 10.16128/j.cnki.1005-3611.2021.01.037

